# Protective Effect of Silymarin against Acrolein-Induced Cardiotoxicity in Mice

**DOI:** 10.1155/2012/352091

**Published:** 2012-12-18

**Authors:** Elahe Taghiabadi, Mohsen Imenshahidi, Khalil Abnous, Fatemeh Mosafa, Mojtaba Sankian, Bahram Memar, Gholamreza Karimi

**Affiliations:** ^1^Department of Phamacodynamy and Toxicology, School of Pharmacy, Mashhad University of Medical Sciences, Mashhad 9177948564, Iran; ^2^Pharmaceutical Research Center, Department of Pharmacodynamy and Toxicology, School of Pharmacy, Mashhad University of Medical Sciences, Mashhad 9177948564, Iran; ^3^Department of Pharmaceutical Biotechnology, Mashhad University of Medical Sciences, Mashhad 9177948564, Iran; ^4^Biotechnology Research Center and School of Pharmacy, Mashhad University of Medical Sciences, Mashhad 9177948564, Iran; ^5^Immunology Research Center, School of Medicine, Mashhad University of Medical Sciences, Mashhad 9177948564, Iran; ^6^Department of Pathology, Imam Reza Hospital, Mashhad University of Medical Sciences, Mashhad 9177948564, Iran; ^7^Medical Toxicology Research Center and School of Pharmacy, Mashhad University of Medical Sciences, Mashhad 9177948564, Iran

## Abstract

Reactive **α**,**β**-unsaturated aldehydes such as acrolein (ACR) are major components of environmental pollutants and have been implicated in the neurodegenerative and cardiac diseases. In this study, the protective effect of silymarin (SN) against cardiotoxicity induced by ACR in mice was evaluated. Studies were performed on seven groups of six animals each, including vehicle-control (normal saline + 0.5% w/v methylcellulose), ACR (7.5 mg/kg/day, gavage) for 3 weeks, SN (25, 50 and 100 mg/kg/day, i.p.) plus ACR, vitamin E (Vit E, 100 IU/kg, i.p.) plus ACR, and SN (100 mg/kg, i.p.) groups. Mice received SN 7 days before ACR and daily thereafter throughout the study. Pretreatment with SN attenuated ACR-induced increased levels of malondialdehyde (MDA), serum cardiac troponin I (cTnI), and creatine kinase-MB (CK-MB), as well as histopathological changes in cardiac tissues. Moreover, SN improved glutathione (GSH) content, superoxide dismutase (SOD), and catalase (CAT) activities in heart of ACR-treated mice. Western blot analysis showed that SN pretreatment inhibited apoptosis provoked by ACR through decreasing Bax/Bcl-2 ratio, cytosolic cytochrome c content, and cleaved caspase-3 level in heart. In conclusion, SN may have protective effects against cardiotoxicity of ACR by reducing lipid peroxidation, renewing the activities of antioxidant enzymes, and preventing apoptosis.

## 1. Introduction

Acrolein (ACR, CH_2_=CH–CHO) has been used as an intermediate for production of some organic chemicals and as a biocide in agricultural and industrial water supply systems [1, 2]. 

According to the Environmental Protection Agency classification, ACR is a high-priority air and water toxic agent [3]. ACR, a ubiquitous environmental pollutant, is produced by incomplete burning of plastic, petrol, wood, gasoline and diesel fuel, paraffin wax, tobacco, and frying of foods in oils [1, 2]. ACR in different levels (10 to 600 *μ*g/kg) has been found in some foods like cheese, donuts, fish, bread, potatoes, and alcoholic beverages [4, 5]. Also, ACR is formed endogenously from the polyunsaturated fatty acids oxidation and the metabolism of polyamine and cyclophosphamide [1, 3]. The sources of ACR that are related to the toxicity and human exposure can be classified into dietary, endogenous, and environmental sources [1]. ACR, a highly reactive *α*,*β*-unsaturated aldehyde, can covalently bind to cell thiols and amine groups in sugars, phospholipids, proteins, and DNA bases and induce oxidative stress and proinflammatory effects in various tissues and cells [6, 7]. ACR provokes its harmful effects through the generation of reactive oxygen species (ROS) and lipid peroxidation in many cell types [8, 9]. Increased evidence indicates that oxidative stress may possess an important effect in the pathogenesis of cardiovascular disorder including ischemic heart disease, heart failure, and atherosclerosis [10–12].

In addition to oxidative stress, ACR can induce apoptosis in different cells by activation of the mitochondrial pathway or death receptor signaling [13, 14]. Some experimental studies indicate that ACR can exert detrimental effects in adult mice cardiomyocytes and heart tissues through apoptotic cell death [6, 9]. Apoptosis has been involved in the toxicity of many chemicals and environmental pollutants [15]. Apoptosis is a physiological cell death that plays a significant role in development and the maintenance of cell homeostasis. An imbalance between apoptotic cell death and cell proliferation damages the normal state and leads to neurodegeneration disorders and heart diseases [14, 16].

Silymarin (SN), a polyphenolic flavonoid, is a standardized extract obtained from the seeds and fruits of milk thistle. It is a mixture of some isomeric flavonolignans including silybin, isosilybin, silydianin, and silychristin [17]. SN indicates effective antioxidant properties [18] in addition to anti-inflammatory [19] and anticarcinogenic actions [20] in animal and human studies. SN has been used clinically to improve chronic inflammatory liver diseases and hepatic cirrhosis. Hepatoprotection can be associated with its antioxidant effects through scavenging free radicals and increasing endogenous antioxidant defenses such as intracellular glutathione (GSH) [21]. It has been demonstrated that SN and milk thistle methanolic extract protect against renal toxicity induced by cisplatin due to the decrease in blood urea nitrogen (BUN), serum creatinine, and tubular damage in rats [22]. It is reported that SN can improve cisplatin-induced increase in serum alanine aminotransferase (ALT) and aspartate aminotransferase (AST), malondialdehyde (MDA), and nitric oxide (NO), as well as the decrease in GSH and the activities of antioxidant enzymes like superoxide dismutase (SOD) and glutathione peroxidase (GSHPx) in liver of rats [23]. SN also attenuates cardiotoxicity and nephrotoxicity induced by adriamycin in rats through the inhibition of lipid peroxidation and GSH depletion in these tissues [24]. Further study indicates that SN can eliminate or significantly decrease the elevation of cytochrome P450 isoform CYP1A1, inducible nitric oxide synthase (iNOS), and metallothionein I-II (MT) expressions in liver, kidney, and heart of the pyridine-treated Syrian hamsters [25]. It has been indicated that pretreatment of male ICR mice with SN protects against doxorubicin-induced oxidative stress and cell death occurring by apoptosis or necrosis in the liver [26]. In addition, SN can considerably ameliorate cisplatin cardiotoxicity by the decrease in serum biochemical marker and MDA level and the increase in GSH content, SOD activity, and the content of total protein [27]. Therefore, we designed the present experiments to evaluate the protective effect of SN against cardiotoxicity induced by ACR subacute exposure in mice.

## 2. Materials and Methods

### 2.1. Chemicals

ACR, SN, phenylmethanesulfonyl fluoride (PMSF), complete protease inhibitor cocktail, and reduced GSH were obtained from Sigma-Aldrich Chemical Company. Vitamin E (Vit E, DL-*α*-Tocopherol acetate) was provided from OSVE Pharmaceutical Co. (Tehran, Iran). Sodium dodecyl sulphate (SDS), MDA, thiobarbituric acid (TBA), N,N,N′,N′-Tetramethylethylenediamine (TEMED), and *β*-Mercaptoethanol (*β*-ME) were provided from Merck. Rabbit monoclonal caspase-3 antibody, rabbit monoclonal caspase-8 antibody, rabbit polyclonal Hsp27 antibodies, rabbit polyclonal Hsp70 antibody, rabbit polyclonal Hsp90 antibody, rabbit monoclonal Bcl-2 antibody, rabbit polyclonal Bax antibody, rabbit polyclonal cytochrome c antibody, anti-rabbit IgG, horseradish peroxidase-conjugated antibody, mouse monoclonal *β*-actin antibody, and anti-mouse IgG, horseradish peroxidase-conjugated antibody were purchased from Cell Signaling. Polyvinylidene fluoride (PVDF) membrane and Bradford protein assay kit were obtained from Bio-Rad. Assay kits for antioxidant enzymes including superoxide dismutase (SOD) and catalase (CAT) were purchased from Biovision (CA, USA), and assay kit for cardiac troponin I (cTnI) was provided from Life Diagnostics, Inc. Assay kit for creatine kinase-MB (CK-MB) was supplied from Pars Azmun (Tehran, Iran). BCA protein assay kit, mitochondria isolation kit for tissue and western blotting detecting reagents (ECL), was supplied from Pierce. All other chemicals were of the highest grade commercially available.

### 2.2. Animal Treatment

Male albino Razi mice (weighing approximately 25–35 g) were obtained from the animal house of the Pharmaceutical Sciences Research Center of Mashhad University of Medical Sciences. Animals were housed in a ventilated room under a 12/12 h light/dark cycle at 21 ± 2°C and had free access to water and food throughout the experimental period. All animal experiments were carried out in accordance with Mashhad University of Medical Sciences, Ethical Committee Acts.

The mice were divided into seven groups of six animals each. The chemicals were administrated in the morning (between 9:00 and 11:00 AM) [28].

*Group 1 (Vehicle-Control Group).* The vehicle-control group received normal saline +0.5% w/v methylcellulose as a vehicle, orally by gavage once a day, for 3 weeks.

*Group 2 (ACR-Treated Group (ACR Group)).* In this group, animals were treated with ACR 7.5 mg/kg per day in normal saline +0.5% w/v methylcellulose, orally by gavage once a day, for 3 weeks.

*Groups 3, 4, and 5 (SN + ACR-Treated Groups (ACR + SN Group)).* SN was dispersed in normal saline +0.5% w/v methylcellulose and was injected at doses 25, 50, and 100 mg/kg per day intraperitoneally to mice, 7 days before ACR (7.5 mg/kg per day, once a day, orally) and daily thereafter throughout the study (3 weeks) [22].

*Group 6 (Vit E + ACR-Treated Group (ACR + Vit E Group)).* Vit E (100 IU/Kg) was dispersed in normal saline +0.5% w/v methylcellulose and, intraperitoneally, was injected three times per week for 3 weeks, and ACR was administrated orally by gavage (7.5 mg/kg per day, once a day) for 3 weeks. Vit E has been considered as a positive control [28].

*Group 7 (SN-Treated Group (SN Group)).* In this group, mice received SN (100 mg/kg per day, intraperitoneally) for 4 weeks.

At the end of the experiment time, after overnight-fasted state, the animals from each group were sacrificed by decapitation. Trunk blood samples were immediately collected in dry tubes and allowed to clot then centrifuged at 2000 ×g for 15 min to separate the sera that were kept at −70°C for biochemical analysis of CK-MB and cTnI levels. Abdomen of each mouse was opened and hearts were carefully removed, washed in ice-cold isotonic saline, immediately immersed in liquid nitrogen, and stored at −80°C for various analyses.

### 2.3. Cardiac Oxidative Stress Assessment

#### 2.3.1. Cardiac Lipid Peroxidation and Reduced GSH Content

For measurement of MDA (as a marker of lipid peroxidation), the heart was homogenized in ice-cold solution of 1.15% KCl to produce 10% homogenate (w/v). The homogenate was centrifuged at 3000 ×g for 10 min, and the obtained supernatant was used as total heart homogenized sample. The levels of protein and MDA were measured in the supernatants. 

The protein content was evaluated using Bradford Protein Assay kit (Bio-Rad Laboratories) and Bovine serum albumin (BSA) as standard. According to the instruction of manufacture, 10 *μ*L of sample supernatants or BSA standards were pipetted into separated microtiter plate wells, and then 200 *μ*L diluted dye reagent (containing 1 part Dye Reagent Concentrate with 4 parts distilled, deionized water) was added to each well and thoroughly mixed on a plate shaker. After 5 minutes incubation at room temperature, the absorbance of reactions was measured at 595 nm. The protein concentration was calculated by the simultaneously prepared calibration curves using BSA standards. 

Lipid peroxidation products were assessed by measuring MDA level according to the method of Niehaus and Samuelsson [29]. The level of MDA in the supernatant was determined spectrophotometrically by measuring thiobarbituric acid-reactive substances with a maximum absorbance at 532 nm. Briefly, 0.5 mL of the sample was mixed with 3 mL of 1% phosphoric acid and 1 mL of 0.6% TBA solution. The mixture was heated in a boiling water bath for 45 min and cooled to the room temperature. Then, 4 mL of n-butanol was added, and mixture was vortexed and centrifuged at 3000 ×g for 10 min. The absorbance of butanol phase (supernatant) was measured at 532 nm. Tissue MDA content was expressed as nmol/mg protein. 

Cardiac GSH content was determined according to the method of Moron et al. [30]. The heart was homogenized in ice cold phosphate buffered saline (PBS), pH 7.4, to obtain 10% homogenate (w/v). The homogenate was centrifuged at 3000 ×g for 10 min, and the collected supernatant was considered as total heart homogenized sample. The contents of protein and GSH were assessed in the supernatants. The protein content was determined using Bradford Protein Assay kit (Bio-Rad Laboratories) and BSA as standard. Reduced GSH content was measured using 5,5′-dithiobis(2-nitrobenzoic acid) (DTNB) which generated a yellow-colored 5-thio-2-nitrobenzoic acid (TNB). In short, equal volumes of sample and 20% trichloroacetic acid (TCA) were mixed and centrifuged at 3000 ×g for 5 min. 0.5 mL of supernatant was added to 2 mL PBS (0.1 M, pH 8.0) and 0.5 mL of 0.04% DTNB reagent. Then, yellow color developed was measured spectrophotometrically at 412 nm. Tissue GSH content was expressed as *μ*g/mg protein.

#### 2.3.2. Cardiac Antioxidant Enzymes

The activity of SOD in heart tissue was determined by available kit provided from Biovision. The heart was homogenized in ice-cold 0.1 M Tris/HCl, pH 7.4 containing 0.5% Triton X-100, 5 mM *β*-ME, 0.1 mg/mL PMSF and centrifuged at 14,000 ×g for 5 minutes at 4°C to obtain supernatant. The contents of protein and SOD activity were assessed in the supernatants. The protein content of obtained supernatants was determined using BCA Protein Assay kit (Pierce) and BSA as standard. According to the instruction of manufacture, 10 *μ*L of sample supernatants or BSA standards was pipetted into separated microtiter plate wells, and then 200 *μ*L BCA Working Reagent (a mixture containing 50 part BCA Reagent A and 1 part BCA Reagent B) was added to each well and carefully mixed on a plate shaker for 30 seconds. After 30 min incubation at 37°C, the absorbance of reactions was measured at 562 nm. The protein concentration was determined by the simultaneously prepared calibration curves using BSA standards. 

In this assay, xanthine and xanthine oxidase generate superoxide anion that reacts with tetrazolium chloride to produce a yellow color formazan dye. SOD activity was measured at 450 nm. The activity of SOD in heart tissue was expressed as (inhibition rate%)/mg protein. 

The activity of CAT was determined using commercially kit obtained from Biovision. To evaluate CAT activity, the heart was homogenized in cold assay buffer and centrifuged at 10,000 ×g for 15 min at 4°C to obtain supernatant. The protein content and CAT activity were measured in the supernatants. The protein content of supernatants was determined using BCA Protein Assay kit (Pierce) and BSA as standard. One unit of CAT was defined as the amount of enzyme needed to decompose 1 *μ*M of H_2_O_2_ in 1 min. The rate of decomposition of H_2_O_2_ was measured spectrophotometrically at 570 nm. The activity of CAT in cardiac tissue was expressed as mU/mg protein. 

### 2.4. Serum Biomarkers of Cardiotoxicity

Serum cTnI was determined using a mice-specific ELISA kit from Life Diagnostics (West Chester, PA), and serum CK-MB was measured using a commercial colorimetric kit from Pars Azmun (Tehran, Iran) according to the instructions of manufacturer.

### 2.5. Histopathological Study

The hearts were fixed in 10% buffered formalin for at least 24 h and then were processed for microscopical assay by a standard protocol. The paraffin sections about 5 *μ*m were stained with hematoxylin and eosin to evaluate under light microscope. Histopathological criteria such as inflammation and edema were determined semiquantitatively from mild (+) to moderate (++), sever (+++), and very sever (++++).

### 2.6. Western Blot Analysis

The heart was homogenized in lysis buffer containing 50 mM Tris-HCl, pH 7.4, 2 mM ethylenediaminetetraacetic acid (EDTA), 2 mM ethylene glycol-bis(2-aminoethylether)-N,N,N′,N′-tetraacetic  acid  (EGTA), 10 mM sodium fluoride, 1 mM sodium orthovanadate, 10 mM *β*-glycerophosphate, 0.2% sodium deoxycholate, 1 mM PMSF, and complete protease inhibitor cocktail. After centrifugation at 10,000 ×g for 10 min, the protein concentration of supernatant was measured using Bradford Protein Assay kit (Bio-Rad Laboratories) and BSA as standard. The supernatant was stored at −80°C. The total of 50 *μ*g of whole protein was used for evaluation of the content of Bax, Bcl-2, caspase-3, caspase-8, Hsp27, Hsp70, and Hsp90.

To assess the level of cytochrome c in cytosolic fraction, the mitochondria isolation kit for tissue (Pierce Biotechnology, Rockford, IL, USA) was used to isolate the cytosolic fraction based on the instruction of manufacturer. Briefly, fresh heart tissues were washed with PBS and were cut to small pieces. Then, BSA/reagent was added, and tissues were homogenized on ice using 40 strokes with dounce tissue grinder. After adding reagent C, homogenate was centrifuged at 700 ×g for 10 min at 4°C. Obtained supernatant was centrifuged at 3000 ×g for 15 min at 4°C, removed from the pellet, and considered as cytosolic fraction. Then, the concentration of protein in cytosolic fraction was determined. The total of 50 *μ*g of the cytosolic fraction protein was used for evaluation of cytochrome c content.

Western blotting was carried out to assess the levels of Bax, Bcl-2, cytochrome c, caspase-3, caspase-8, Hsp27, Hsp70, and Hsp90 by appropriate antibodies, followed by a horseradish peroxidase-conjugated secondary antibody. Proteins (50 *μ*g) were solubilised in Laemmli sample buffer. Then, the samples were boiled for 5 min at 95°C, and the denatured samples were separated by 12% SDS-polyacrylamide gel electrophoresis and transferred to PVDF membrane. Following the transfer, the membranes were blocked by overnight incubation at 4°C in a 5% skim milk. After blocking, blots were incubated with antibodies: Bax (Cell Signaling no. 2772), Bcl-2 (Cell Signaling no. 2870), cytochrome c (Cell Signaling no. 4272), caspase-3 (Cell Signaling no. 9665), caspase-8 (Cell Signaling no. 4790), Hsp27 (Cell Signaling no. 2442), Hsp70 (Cell Signaling no. 4872), and Hsp90 (Cell Signaling no. 4874) at 1000-fold dilutions for 2 h at room temperature. Mouse monoclonal *β*-actin antibody (Cell Signaling no. 3700S) was used to confirm equal loading conditions. The blots were washed three times with TBST (TBS with 0.1% Tween 20) and then incubated with horseradish peroxidase-conjugated anti-rabbit antibody (no. 7074 Cell Signaling) or horseradish peroxidase-conjugated anti-mouse antibody (no. 7076S Cell Signaling) at 1 : 3000 dilution for 1 h at room temperature, again followed by three washes with TBST. Finally, protein bands were detected using enhanced chemiluminescnce (ECL) reagent (Pierce ECL western blotting substrate) and Alliance 4.7 Geldoc (UK). Protein bands were analyzed using UVtec software (UK). The protein levels were normalized against *β*-actin intensity.

### 2.7. Statistical Analysis

The mean ± SEM was determined for each study group and tested with analysis of variance followed by the multiple comparison test of Tukey-Kramer. Discrepancies with *P* < 0.05 were considered significant.

## 3. Results

### 3.1. Effect of SN on Lipid Peroxidation and GSH Content

**Figure 1:**
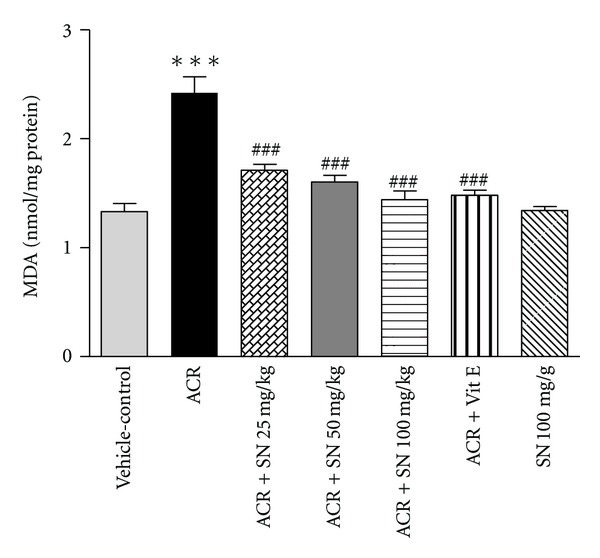
The effect of ACR and SN pretreatment on MDA level in heart of mice. Vehicle-control group received normal saline +0.5% w/v methylcellulose, orally for 3 weeks. ACR was given (7.5 mg/kg, orally) for 3 weeks. SN was injected 7 days before ACR and daily thereafter throughout the study (3 weeks). Vit E (100 IU/Kg, three times per week) plus ACR were administrated for 3 weeks. SN alone at dose (100 mg/kg, intraperitoneally) was treated for 4 weeks. Data are expressed as mean ± SEM. ***P < 0.001 versus vehicle-control group, ^###^ *P* < 0.001 versus ACR group, Tukey-Kramer test, *n* = 6.

**Figure 2:**
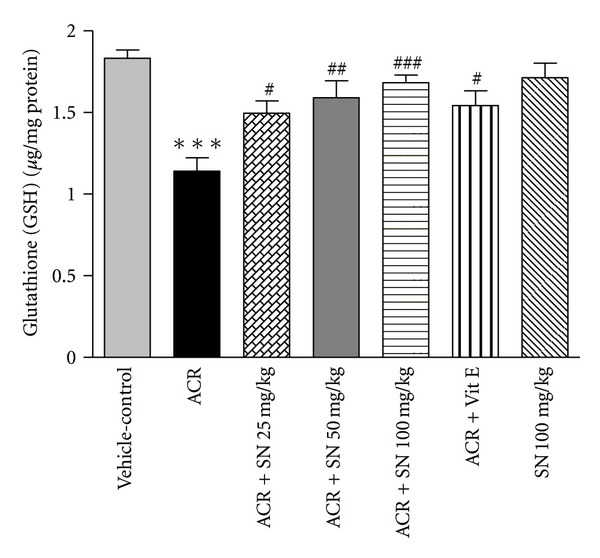
The effect of ACR and SN pretreatment on GSH content in heart of mice. Vehicle-control group received normal saline +0.5% w/v methylcellulose, orally for 3 weeks. ACR was given (7.5 mg/kg, orally) for 3 weeks. SN was injected 7 days before ACR and daily thereafter throughout the study (3 weeks). Vit E (100 IU/Kg, three times per week) plus ACR were administrated for 3 weeks. SN alone at dose (100 mg/kg, intraperitoneally) was treated for 4 weeks. Data are expressed as mean ± SEM. ***P < 0.001 versus vehicle-control group, ^###^ *P* < 0.001, ^##^ *P* < 0.01, and ^#^ *P* < 0.05 versus ACR group, Tukey-Kramer test, *n* = 6.

Oxidative stress can be evaluated by assessment of the end products of oxidative damage such as MDA that indicated the lipid peroxidation of cell membrane and the content of GSH, which scavenges free radicals and detoxifies different xenobiotics [31]. The level of MDA in the cardiac tissue was shown in Figure 1. Cardiac level of MDA was significantly increased in ACR-treated mice compared to vehicle-control (*P* < 0.001). Pretreatment with SN in ACR + SN groups resulted in a significant decrease in MDA value compared to ACR-treated group (*P* < 0.001). SN alone at dose 100 mg/Kg did not increase MDA level compared to vehicle-control group. The content of GSH in heart was shown in Figure 2. In ACR-treated mice, cardiac content of GSH was significantly reduced compared to vehicle-control (*P* < 0.001). However, pretreatment with SN in ACR + SN groups significantly increased the content of GSH compared to ACR-treated mice (*P* < 0.05). The result of this study indicated that treatment of mice with SN alone at dose 100 mg/Kg did not change the content of GSH compared to vehicle-control group.

### 3.2. Effect of SN on Cardiac Antioxidant Enzymes

**Figure 3:**
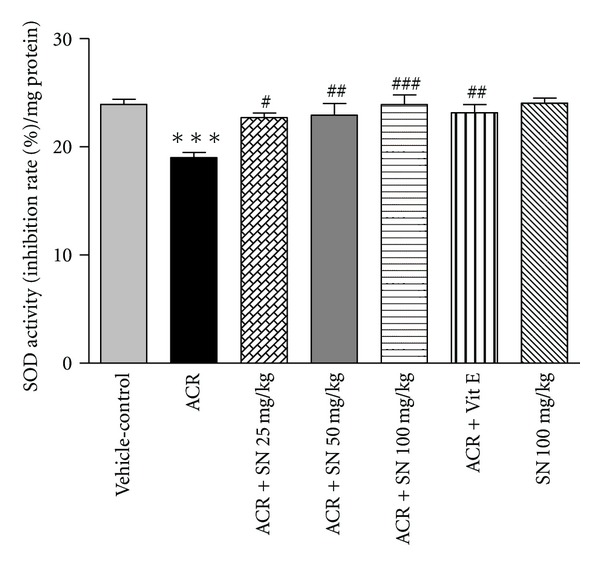
The effect of ACR and SN pretreatment on SOD activity in heart of mice. Vehicle-control group received normal saline +0.5% w/v methylcellulose, orally for 3 weeks. ACR was given (7.5 mg/kg, orally) for 3 weeks. SN was injected 7 days before ACR and daily thereafter throughout the study (3 weeks). Vit E (100 IU/Kg, three times per week) plus ACR were administrated for 3 weeks. SN alone at dose (100 mg/kg, intraperitoneally) was treated for 4 weeks. Data are expressed as mean ± SEM. ***P < 0.001 versus vehicle-control group, ^###^ *P* < 0.001, ^##^ *P* < 0.01, and ^#^ *P* < 0.05 versus ACR group, Tukey-Kramer test, *n* = 6.

**Figure 4:**
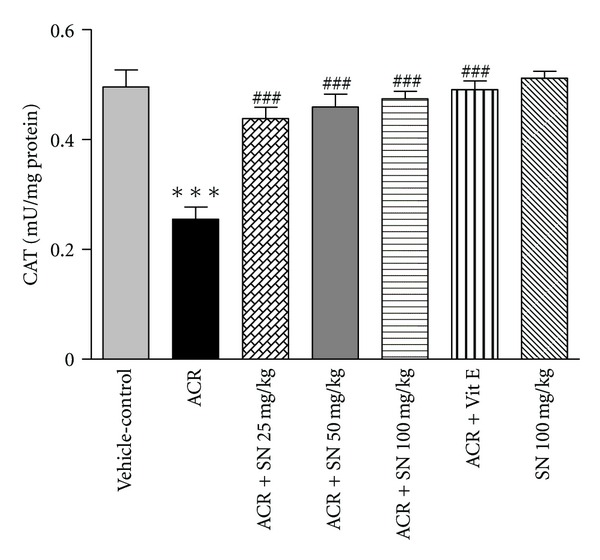
The effect of ACR and SN pretreatment on CAT activity in heart of mice. Vehicle-control group received normal saline +0.5% w/v methylcellulose, orally for 3 weeks. ACR was given (7.5 mg/kg, orally) for 3 weeks. SN was injected 7 days before ACR and daily thereafter throughout the study (3 weeks). Vit E (100 IU/Kg, three times per week) plus ACR were administrated for 3 weeks. SN alone at dose (100 mg/kg, intraperitoneally) was treated for 4 weeks. Data are expressed as mean ± SEM. ***P < 0.001 versus vehicle-control group, ^###^ *P* < 0.001 versus ACR group, Tukey-Kramer test, *n* = 6.

The antioxidant enzymes SOD and CAT are members of endogenous antioxidants that play an important role in protection of the biological systems against oxidative damages [31]. The activities of SOD and CAT in cardiac tissue were demonstrated in Figures 3 and 4, respectively. Administration of ACR to mice significantly reduced the cardiac activities of SOD and CAT as compared to the vehicle-control (*P* < 0.001). However, a significant increase in the activities of SOD and CAT was observed upon pretreatment with SN in ACR + SN groups compared to ACR-treated group (*P* < 0.05 and *P* < 0.001, resp.). Data showed that treatment with SN alone did not decrease the cardiac activities of SOD and CAT compared to the vehicle-control.

### 3.3. Effect of SN on Serum Biomarkers and Histopathological Study

**Figure 5:**
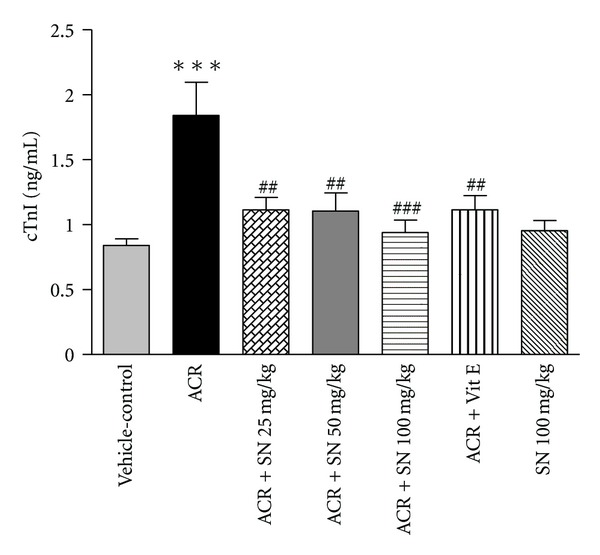
The effect of ACR and SN pretreatment on serum cTnI level. Vehicle-control group received normal saline +0.5% w/v methylcellulose, orally for 3 weeks. ACR was given (7.5 mg/kg, orally) for 3 weeks. SN was injected 7 days before ACR and daily thereafter throughout the study (3 weeks). Vit E (100 IU/Kg, three times per week) plus ACR were administrated for 3 weeks. SN alone at dose (100 mg/kg, intraperitoneally) was treated for 4 weeks. Data are expressed as mean ± SEM. ***P < 0.001 versus vehicle-control group, ^###^ *P* < 0.001 and ^##^ *P* < 0.01 versus ACR group, Tukey-Kramer test, *n* = 6.

**Figure 6:**
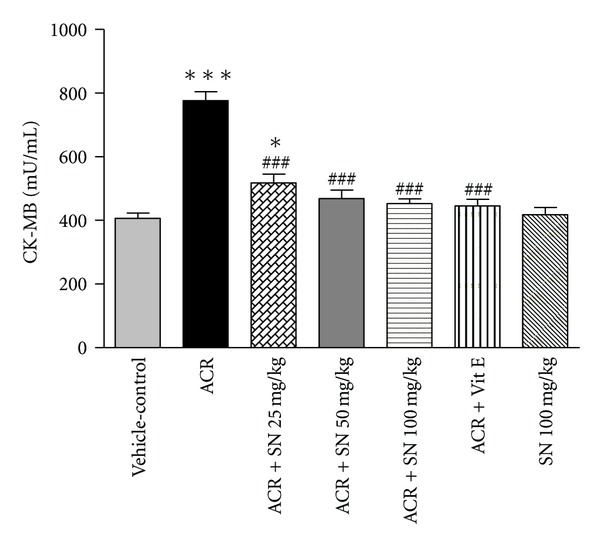
The effect of ACR and SN pretreatment on serum CK-MB level. Vehicle-control group received normal saline +0.5% w/v methylcellulose, orally for 3 weeks. ACR was given (7.5 mg/kg, orally) for 3 weeks. SN was injected 7 days before ACR and daily thereafter throughout the study (3 weeks). Vit E (100 IU/Kg, three times per week) plus ACR were administrated for 3 weeks. SN alone at dose (100 mg/kg, intraperitoneally) was treated for 4 weeks. Data are expressed as mean ± SEM. ***P < 0.001 and *P < 0.05 versus vehicle-control group, ^###^ *P* < 0.001 versus ACR group, Tukey-Kramer test, *n* = 6.

cTnI and CK-MB are released from damaged myocytes, and these are sensitive factors of myocardial damages [32]. Assessment of cTnI and CK-MB levels in serum was shown in Figures 5 and 6, respectively. The present data revealed that the levels of cTnI and CK-MB in serum were significantly increased in ACR-treated mice as compared to vehicle-control (*P* < 0.001). Pretreatment with SN in ACR + SN groups significantly decreased these biomarkers compared to ACR-treated group (*P* < 0.01 and *P* < 0.001, resp.). The levels of cTnI and CK-MB in serum showed no significant changes in mice treated with SN alone compared to the vehicle-control. 

**Figure 7:**
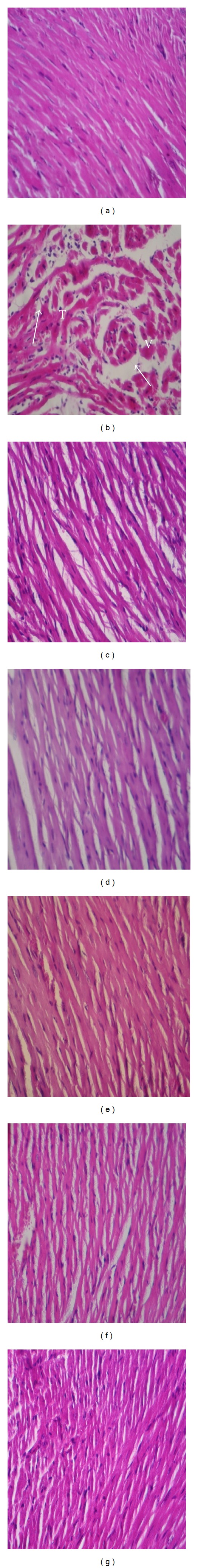
Cardiac tissues of vehicle-control mice show normal histological pattern. Hematoxylin and eosin ×400. (a) ACR-treated mice indicate prominent interstitial edema (V) and focal moderate interstitial inflammation (T) in heart tissues. Hematoxylin and eosin ×400. (b) Cardiac sections of mice treated with SN (25, 50, and 100 mg/Kg, i.p.) plus ACR and Vit E plus ACR demonstrate the decrease in damages. Hematoxylin and eosin ×400. ((c), (d), (e), and (f), resp.) Cardiac tissues of animals which were injected with SN (100 mg/Kg) show normal myocardium architecture. Hematoxylin and eosin ×400. (g) Vehicle-control group received normal saline +0.5% w/v methylcellulose, orally for 3 weeks. ACR was given (7.5 mg/kg, orally) for 3 weeks. SN was injected 7 days before ACR and daily thereafter throughout the study (3 weeks). Vit E (100 IU/Kg, three times per week) plus ACR were administrated for 3 weeks. SN alone at dose (100 mg/kg, intraperitoneally) was treated for 4 weeks.

Cardiotoxicity induced by ACR was further evaluated by hematoxylin and eosin stained sections. Hearts from vehicle-control group and SN alone at dose (100 mg/Kg) indicated normal myocardium architecture (Figures 7(a) and 7(g)). As shown in Figure 7(b), histological findings indicated abnormalities and toxic effects in cardiac tissues of mice exposed to ACR. Prominent interstitial edema (+++) and focal moderate interstitial inflammation (++) were observed in heart of ACR-treated mice. Pretreatment with SN in ACR + SN groups reduced myocardial damages (Figures 7(c), 7(d), 7(e), and 7(f), resp.).

### 3.4. Effect of SN on Cardiac Apoptosis by Western Blot Analysis

**Figure 8:**
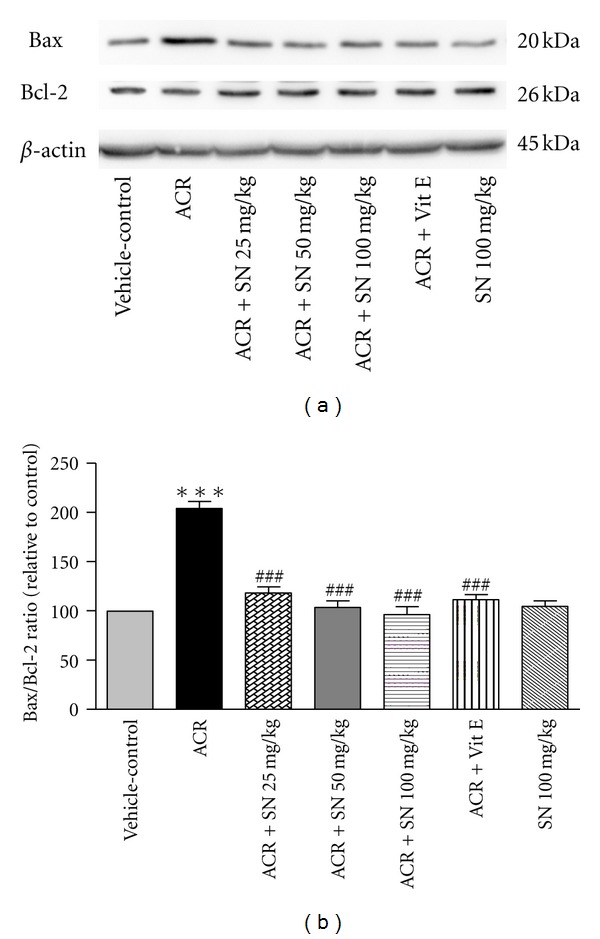
The effect of ACR and SN pretreatment on the protein levels of Bax and Bcl-2 in heart of mice. (a) Representative western blots indicating specific bands for Bax, Bcl-2, and *β*-actin as an internal control. Equal amounts of protein sample (50 *μ*g) obtained from whole heart homogenate were applied in each lane. These bands are representative of six separate experiments. (b) Densitometric data of protein analysis. Vehicle-control group received normal saline +0.5% w/v methylcellulose, orally for 3 weeks. ACR was given (7.5 mg/kg, orally) for 3 weeks. SN was injected 7 days before ACR and daily thereafter throughout the study (3 weeks). Vit E (100 IU/Kg, three times per week) plus ACR were administrated for 3 weeks. SN alone at dose (100 mg/kg, intraperitoneally) was treated for 4 weeks. Data are expressed as the mean ± SEM. ***P < 0.01 versus vehicle-control group, ^###^ *P* < 0.001 versus ACR group.

**Figure 9:**
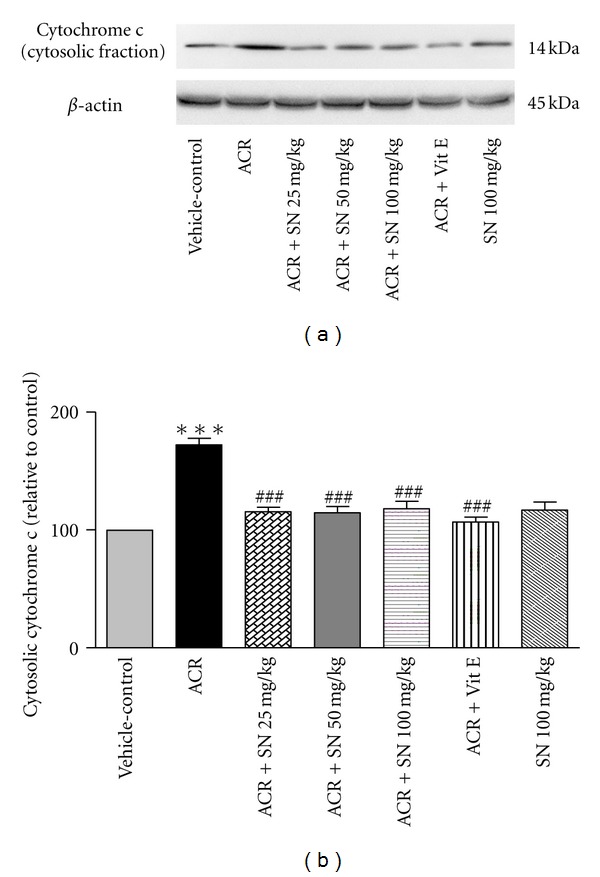
The effect of ACR and SN pretreatment on the protein level of cytochrome c in cytosolic fraction in heart of mice. (a) Representative western blots indicating specific bands for cytochrome c and *β*-actin as an internal control. Equal amounts of protein sample (50 *μ*g) obtained from the cytosolic fraction homogenate were applied in each lane. These bands are representative of six separate experiments. (b) Densitometric data of protein analysis. Vehicle-control group received normal saline +0.5% w/v methylcellulose, orally for 3 weeks. ACR was given (7.5 mg/kg, orally) for 3 weeks. SN was injected 7 days before ACR and daily thereafter throughout the study (3 weeks). Vit E (100 IU/Kg, three times per week) plus ACR were administrated for 3 weeks. SN alone at dose (100 mg/kg, intraperitoneally) was treated for 4 weeks. Data are expressed as the mean ± SEM. ***P < 0.01 versus vehicle-control group, ^###^ *P* < 0.001 versus ACR group.

**Figure 10:**
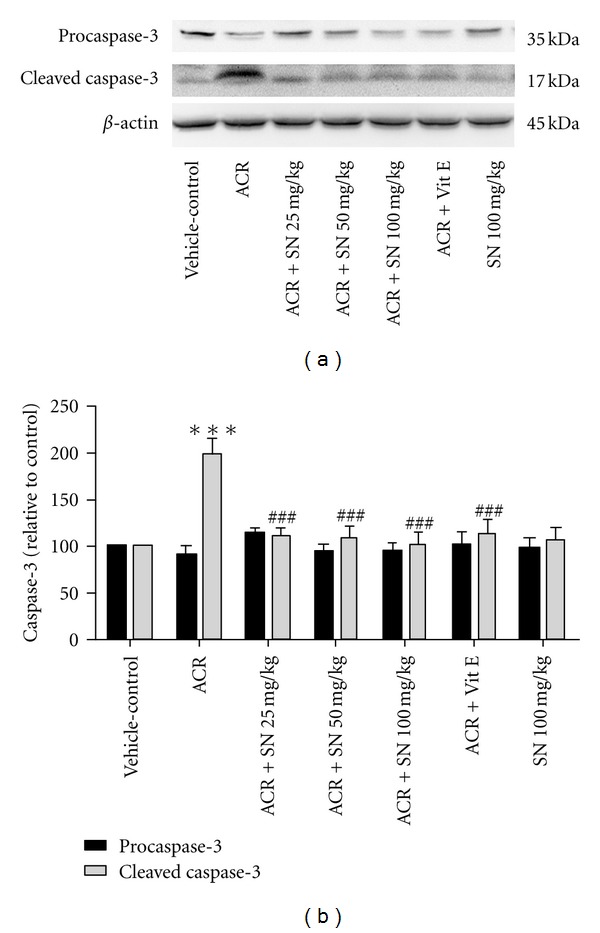
The effect of ACR and SN pretreatment on the protein level of caspase-3 (pro- and cleaved caspase-3) in heart of mice. (a) Representative western blots indicating specific bands for procaspase-3, cleaved caspase-3, and *β*-actin as an internal control. Equal amounts of protein sample (50 *μ*g) obtained from whole heart homogenate were applied in each lane. These bands are representative of six separate experiments. (b) Densitometric data of protein analysis. Vehicle-control group received normal saline +0.5% w/v methylcellulose, orally for 3 weeks. ACR was given (7.5 mg/kg, orally) for 3 weeks. SN was injected 7 days before ACR and daily thereafter throughout the study (3 weeks). Vit E (100 IU/Kg, three times per week) plus ACR were administrated for 3 weeks. SN alone at dose (100 mg/kg, intraperitoneally) was treated for 4 weeks. Data are expressed as the mean ± SEM. ***P < 0.01 versus vehicle-control group, ^###^ *P* < 0.001 versus ACR group.

**Figure 11:**
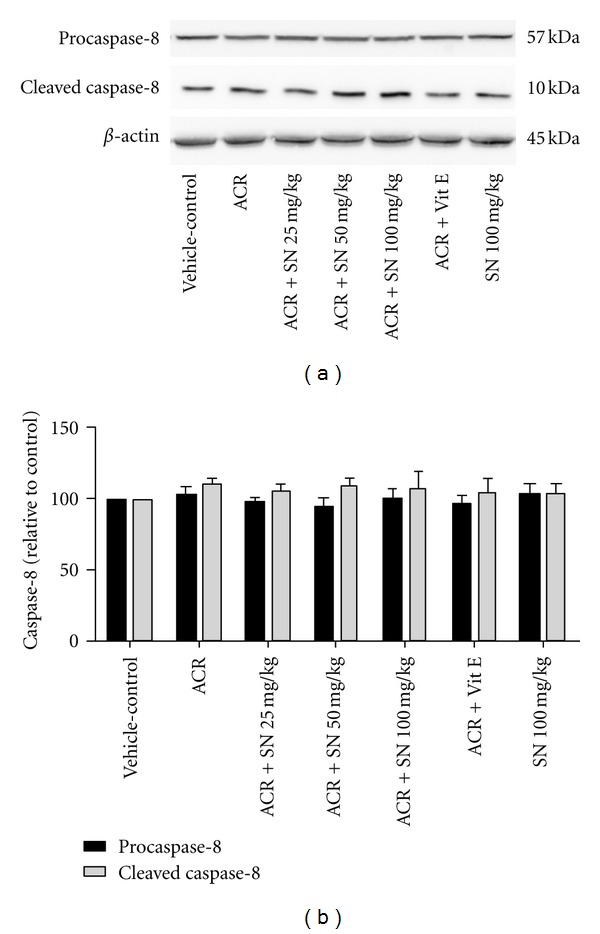
The effect of ACR and SN pretreatment on the protein level of caspase-8 (pro- and cleaved caspase-8) in heart of mice. (a) Representative western blots indicating specific bands for procaspase-8, cleaved caspase-8, and *β*-actin as an internal control. Equal amounts of protein sample (50 *μ*g) obtained from whole heart homogenate were applied in each lane. These bands are representative of six separate experiments. (b) Densitometric data of protein analysis. Vehicle-control group received normal saline +0.5% w/v methylcellulose, orally for 3 weeks. ACR was given (7.5 mg/kg, orally) for 3 weeks. SN was injected 7 days before ACR and daily thereafter throughout the study (3 weeks). Vit E (100 IU/Kg, three times per week) plus ACR were administrated for 3 weeks. SN alone at dose (100 mg/kg, intraperitoneally) was treated for 4 weeks. Data are expressed as the mean ± SEM. *P* > 0.05.

**Figure 12:**
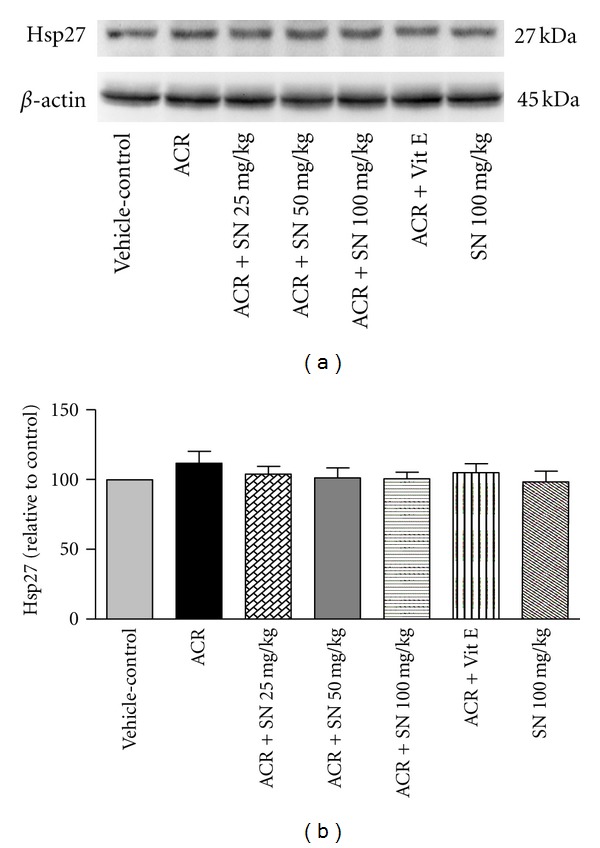
The effect of ACR and SN pretreatment on the protein level of Hsp27 in heart of mice. (a) Representative western blots indicating specific bands Hsp27 and *β*-actin as an internal control. Equal amounts of protein sample (50 *μ*g) obtained from whole heart homogenate were applied in each lane. These bands are representative of six separate experiments. (b) Densitometric data of protein analysis vehicle-control group received normal saline +0.5% w/v methylcellulose, orally for 3 weeks. ACR was given (7.5 mg/kg, orally) for 3 weeks. SN was injected 7 days before ACR and daily thereafter throughout the study (3 weeks). Vit E (100 IU/Kg, three times per week) plus ACR were administrated for 3 weeks. SN alone at dose (100 mg/kg, intraperitoneally) was treated for 4 weeks. Data are expressed as the mean ± SEM. *P* > 0.05.

**Figure 13:**
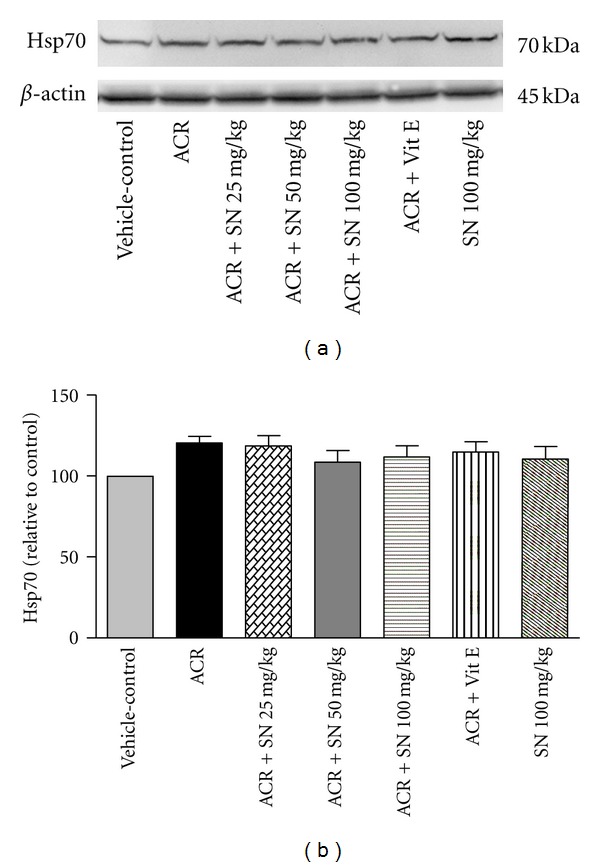
The effect of ACR and SN pretreatment on the protein level of Hsp70 in heart of mice. (a) Representative western blots indicating specific bands for Hsp70 and *β*-actin as an internal control. Equal amounts of protein sample (50 *μ*g) obtained from whole heart homogenate were applied in each lane. These bands are representative of six separate experiments. (b) Densitometric data of protein analysis. Vehicle-control group received normal saline +0.5% w/v methylcellulose, orally for 3 weeks. ACR was given (7.5 mg/kg, orally) for 3 weeks. SN was injected 7 days before ACR and daily thereafter throughout the study (3 weeks). Vit E (100 IU/Kg, three times per week) plus ACR were administrated for 3 weeks. SN alone at dose (100 mg/kg, intraperitoneally) was treated for 4 weeks. Data are expressed as the mean ± SEM. *P* > 0.05.

**Figure 14:**
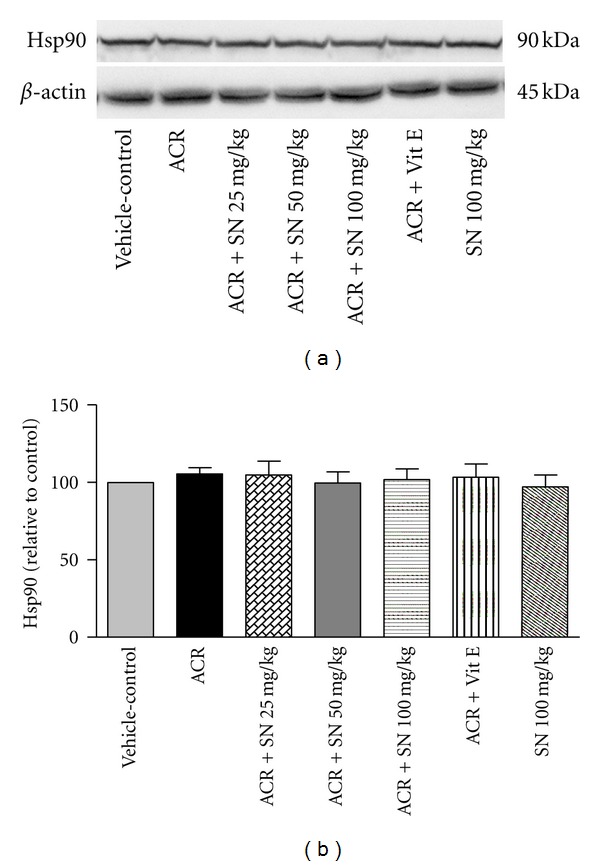
The effect of ACR and SN pretreatment on the protein level of Hsp90 in heart of mice. (a) Representative western blots indicating specific bands for Hsp90 and *β*-actin as an internal control. Equal amounts of protein sample (50 *μ*g) obtained from whole heart homogenate were applied in each lane. These bands are representative of six separate experiments. (b) Densitometric data of protein analysis vehicle-control group received normal saline +0.5% w/v methylcellulose, orally for 3 weeks. ACR was given (7.5 mg/kg, orally) for 3 weeks. SN was injected 7 days before ACR and daily thereafter throughout the study (3 weeks). Vit E (100 IU/Kg, three times per week) plus ACR were administrated for 3 weeks. SN alone at dose (100 mg/kg, intraperitoneally) was treated for 4 weeks. Data are expressed as the mean ± SEM. *P* > 0.05.

The cardiac level of proteins involved in apoptosis pathway was shown in Figures 8–14. 

The Bcl-2 family of proteins is considered to reside in the mitochondrial outer membrane and is involved in apoptosis pathway by modulating the membrane permeability. Bax and BcL-2 are two members of the Bcl-2 family that possess an important effect in the regulation of apoptosis. To assess the protective effect of SN on ACR-induced apoptosis in heart of mice, the Bax/Bcl-2 ratio was evaluated [16, 33]. As shown in Figures 8(a) and 8(b), administration of ACR to mice significantly upregulated the Bax/Bcl-2 ratio compared to vehicle-control (*P* < 0.001), and pretreatment with SN in ACR + SN groups significantly attenuated the Bax/Bcl-2 ratio compared to ACR-treated mice (*P* < 0.001). The current data showed that treatment of mice with SN alone did not upregulate the Bax/Bcl-2 ratio in heart tissues compared to the vehicle-control.

The libration of cytochrome c from the mitochondria to the cytoplasm leads to activation of the caspases involved in intrinsic apoptotic pathway, and also cytochrome c is considered one of the upstream signals for induction of caspase-3 [16, 34]. The western blot analysis was conducted to evaluate the effects of ACR and SN on the cardiac level of cytochrome c in cytosolic fraction. As shown in Figures 9(a) and 9(b), the content of cytochrome c in cytosolic fraction was significantly increased in ACR-treated animals as compared to vehicle-control group (*P* < 0.001). However, pretreatment with SN in ACR + SN groups exerted a significant decrease in the content of cytosolic cytochrome c as compared to ACR-treated mice (*P* < 0.001). SN alone did not change the content of cytochrome c in the cytosolic fraction compared to vehicle-control. 

Caspase-3 is an important effector caspase (the executioner caspase) which can be activated during the apoptotic pathway [35]. To assess the effects of ACR and SN on the activation of caspase-3, western blot analysis was conducted to determine the level of cleaved caspase-3 and procaspase-3 in heart tissues. Figures 10(a) and 10(b) indicated that administration of ACR to mice significantly increased the level of cleaved caspase-3 as compared to vehicle-control (*P* < 0.001), and pretreatment with SN in ACR + SN groups significantly decreased the level of cleaved caspase-3 compared to ACR-treated animals (*P* < 0.001). Also, data showed that the level of cleaved caspase-3 was alleviated in ACR + Vit E group. SN treatment alone at dose 100 mg/Kg did not increase the level of cleaved caspase-3 compared to vehicle-control in heart tissue.

The extrinsic pathway of apoptosis is mediated by death receptors that lead to activation of the initiator caspase-8 [36]. To examine the effects of ACR and SN on the activation of caspase-8, western blot analysis was performed to determine the expression of procaspase-8 and cleaved caspase-8 in cardiac tissues. As shown in Figures 11(a) and 11(b), administration of ACR to animals indicated no significant change in the level of cleaved caspase-8 compared to the vehicle-control (*P* > 0.05), and the levels of cleaved caspase-8 were not different significantly in all groups (*P* > 0.05).

Heat shock proteins (Hsps) are considered to have the beneficial effects in the biological systems. In response to several stimuli like oxidative stress, the expression of Hsps is increased, and some of them such as Hsp27, Hsp70, and Hsp90 can protect cells against oxidative damages and apoptosis [37]. To investigate the effects of ACR and SN on the levels of Hsp27, Hsp70, and Hsp90, western blot analysis was performed to detect the levels of these proteins in heart of mice. As shown in Figures 12(a), 12(b), 13(a), 13(b), 14(a), and 14(b), no significant changes in the levels of Hsp27, Hsp70, and Hsp90 were observed in ACR-treated mice compared to the vehicle-control, and the levels of these proteins were not different significantly in all groups (*P* > 0.05). 

## 4. Discussion

Silymarin, a polyphenolic flavonoid, is isolated from the seeds and fruits of milk thistle. Antioxidant and antiapoptotic effects of SN have been reported in many studies [17, 21, 23, 24]. ACR is a ubiquitous environmental pollutant and has important relevance to public health. It is reported that large amounts of ACR are present in foods, cigarette smoke, water, heated oils, automobile exhaust, and coal [2]. Some studies reported that the detrimental and cardiotoxic effect of ACR is related to the generation of ROS and lipid peroxidation [8, 9]. Oxidative stress and peroxidation of lipid have an important role in many disorders like heart diseases [12]. The main objective of this study was to investigate the protective effects of SN against ACR-induced cardiotoxicity in mice.

The results showed that cardiac level of MDA was significantly increased, while content of GSH was significantly decreased following ACR administration as compared to vehicle-control group. Oxidative stress induces the lipid peroxidation, and that is due to the interaction between free radicals of diverse origin and cell membrane unsaturated fatty acids. This condition leads to accumulation of several toxic products and MDA. The level of MDA is considered to be an appropriate indicator of lipid peroxidation [26]. These data demonstrated that ACR induced oxidative stress and lipid proxidation of membrane unsaturated fatty acids. Our results are in agreement with other studies which reported that ACR can induce cardiotoxicity due to oxidative stress and rapid depletion of GSH [6, 9]. The lipid peroxidation induced by ACR may be attributed to the generation of oxygen free radicals. It is possible that some mechanisms are involved. First, ACR can be metabolized to superoxide by xanthine oxidase or aldehyde dehydrogenase. Second, ACR increased ROS production by rapid depletion of endogenous antioxidants, like GSH [38, 39]. Also, cardiac tissue is very sensitive to oxidative damages due to its highly oxidative metabolism and lower antioxidant systems [24]. Data indicated that administration of SN significantly protected against ACR-induced cardiotoxicity through the inhibition of lipid peroxidation and the increase in GSH content. Several investigations have demonstrated the antioxidant properties of SN in different models. In some studies, it was shown that SN could attenuate lipid peroxidation induced by doxorubicin in heart, liver, and kidney [24, 26]. Also, SN can improve the cisplatin-induced cardiotoxicity including the oxidative stress and decrease in GSH content [27]. The protective effect of SN may be related to antioxidant activities because of its effects as lipid peroxidation inhibitor and plasma membrane stabilizer and its stimulation for antioxidants [24, 27]. In addition, antioxidants containing thiol groups like reduced GSH are one of the important cellular defenses against free radicals and oxidative stress. The content of reduced GSH and total thiols are a marker of oxidative damage in heart tissue. GSH acts as scavenger of free radicals and eliminates several xenobiotics [31].

According to data, SN pretreatment ameliorated ACR toxicity by increasing of GSH content in cardiac tissues. However, the content of GSH in the heart was not changed after treatment of mice with SN alone at dose 100 mg/Kg compared to vehicle-control, and our results are in agreement with other reports which indicate that treatment with SN alone has not changed the level of GSH in liver, heart, and kidney [23, 24, 40]. 

Current data showed that the activities of the cardiac antioxidant enzymes CAT and SOD were significantly decreased in response to ACR administration compared to vehicle-control group. These findings supported the previously observed GSH depletion and MDA accumulation in heart tissues. The deleterious effect of ACR on the activities of CAT and SOD has been reported in some tissues and cell types [41, 42]. The decrease in SOD and CAT activities may be related to the elevation of superoxide radical generation during ACR metabolism. Antioxidant enzymes such as SOD and CAT are implicated to be the first line of biological defense against oxidative stress [31]. The SOD transforms superoxide radicals into hydrogen peroxide and O_2_, and CAT converts hydrogen peroxide into H_2_O and O_2_. Therefore, SOD and CAT play an important role in the elimination of ROS [43]. The reduction in the activities of antioxidant enzymes can be elucidated on the basis of their exhaustion against oxidative damages [44]. 

It is reported that neuroprotective effect of SN may be due to improvement in antioxidant defense system in focal ischemic rats [45]. In addition, SN can increase antioxidant enzyme activity in liver fibrosis induced by thioacetamide in rats [46]. Our results showed that SN pretreatment can improve the antioxidant enzymes activities in heart tissues. 

ACR administration to mice significantly increased serum cTnI and CK-MB levels compared to the vehicle-control. The increased level of cTnI and CK-MB in ACR-treated group can be attributed to cardiac damage, and this is confirmed by the pathologic findings of this organ. In contrast, SN pretreatment significantly inhibited ACR-induced elevation in serum levels of cTnI and CK-MB. In fact, cTnI is considered to be a very sensitive, specific, and persistent marker of myocardial injury, and CK-MB is one of the available indicators for the diagnosis of heart damage [47]. Our results are in consonance with previous study which reported that SN improved cisplatin-induced cardiotoxicity by decreasing the levels of serum CK-MB and plasma cTnI [27]. The protective effect of SN is associated with inhibition of lipid peroxidation activity that leads to stabilizing of plasma membranes and prevents the release of cardiac enzymes. Also, histopathological findings indicated that SN pretreatment protected against ACR-induced toxicity due to the decrease of cardiac damages in these animals. Therefore, the reduction in leakage of these indicators may be related to stability of the cardiomyocyte integrity.

ACR-induced apoptosis in cardiac tissue was assessed by western blot analysis of proteins involved in apoptotic pathway. The results showed that ACR induced apoptosis through the increase in the Bax/Bcl-2 ratio which caused the elevation of cytochrome c in the cytoplasm. The increase in the cytosolic fraction of cytochrome c activated caspase-3 and raised the level of cleaved caspase-3 which led to apoptosis induction in cardiac tissues. However, the results exerted no significant change in the level of cleaved caspase-8 in ACR-treated group compared to the vehicle-control. Other study indicated that ACR toxicity may be partly due to the induction of apoptosis. It has been reported that ACR causes apoptosis in A549 human lung cells through the mitochondrial pathway which may be mediated by the reactive oxygen species [35]. ACR can provoke apoptosis in the adult mice cardiomyocytes by increasing intracellular oxygen free radicals and calcium concentration [9]. Also, it has been demonstrated that long-term oral treatment of ACR induces oxidative stress and apoptosis in the heart of mice [6]. 

It is known that oxidative stress and the decrease in antioxidant capacity of cells may be an important mediator of apoptotic cell death [48, 49]. Also, cardiac muscle cells after differentiation have lost their proliferative activity, and apoptotic inducer can considerably damage this organ [27]. The mitochondrial and death receptor pathways are two main apoptotic processes in biological systems [16]. The balance between pro- and antiapoptotic proteins of the Bcl-2 family plays a crucial role in progression of apoptosis, so the ratio of Bax/Bcl-2 and libration of cytochrome c from mitochondria to the cytoplasm are considered in assessment of apoptosis [33, 50]. Indeed, the release of cytochrome c from the mitochondria to the cytosol induces the activation of caspase-3 in cells [16]. Caspase-8, the initiator caspase, may be induced by cell surface death receptors. Administration of ACR to mice indicated no significant alteration in caspase-8 activity, so the extrinsic pathway of apoptosis might have a minor effect in the toxicity of ACR in cardiac tissues of mice. While ACR can activate the mitochondrial pathway of apoptosis through the increase in Bax/Bcl-2 ratio and the elevation of cytochrome c in cytoplasm followed by an increase in the level of cleaved caspase-3 in heart of ACR-treated group, it is reported that the mitochondrial pathway of apoptosis is induced by several factors such as oxidative stress and exposure to toxic substances. Therefore, ACR-induced mitochondrial pathway of apoptosis may be mediated by generation of oxidants and reactive oxygen species, decrease in antioxidant capacity, and finally induction of oxidative stress [35]. 

According to data, SN can attenuate apoptosis induced by ACR in heart of mice. Administration of SN reduced the ratio of Bax/Bcl-2 and cytochrome c level in cytosolic fraction. The reduction of the cytosolic cytochrome c decreased the activation of caspase-3 which led to antiapoptotic effect of SN in cardiac tissue. These results suggested that SN could alleviate cardiac injury caused by ACR through suppressing mitochondrial pathway of apoptosis. Our results are in agreement to other studies that reported antiapoptotic properties of SN against toxicity and detrimental effects of cisplatin, doxorubicin, and lead on heart and liver tissues [26, 27, 51].

The results showed that the levels of Hsp27, Hsp70, and Hsp90 did not reveal significant changes in ACR-treated mice compared to the vehicle-control. The cytoprotective activities of Hsps have been indicated in some pieces of research especially in vitro; however, these effects have not always been observed in vivo studies [52]. In the condition that cell death occurs within a short period, this may not allow time for the effective delivery of Hsps induces to damage sites or fast rearrangement of Hsps to exert their protective roles in a timely manner [37, 52]. It is possible that the level of Hsps has changed during the treatment protocol, but at the time of sampling, we could not observe the increased level of them. It seems that expression of Hsps depends on many factors such as time of sampling, tissue types, severity of cell injury, stress inducer, and protective mediator [53]. 

## 5. Conclusion

Our data indicate that SN has protective effects against ACR-induced cardiotoxicity in mice through attenuating lipid peroxidation, increasing GSH content, renewing the activities of antioxidant enzymes, and alleviating apoptosis by modulating Bax/Bcl-2 ratio, the cytosolic cytochrome c content, and the level of cleaved caspase-3.

## Abbreviations

ACR: Acrolein
SN: Silymarin
Vit E: Vitamin E
MDA: Malondialdehyde
cTnI: Cardiac troponin I
CK-MB: Creatine kinase-MB
GSH: Glutathione
SOD: Superoxide dismutase
CAT: Catalase
ROS: Reactive oxygen species
BUN: Blood urea nitrogen
ALT: Alanine aminotransferase
AST: Aspartate aminotransferase
NO: Nitric oxide
GSHPx: Glutathione peroxidase
iNOS: Inducible nitric oxide synthase
MT: Metallothionein I-II
PMSF: Phenylmethanesulfonyl fluoride
SDS: Sodium dodecyl sulphate
TEMED: N,N,N′,N′-Tetramethylethylenediamine
*β*-ME: *β*-Mercaptoethanol
TBA: Thiobarbituric acid
PVDF: Polyvinylidene fluoride
BSA: Bovine serum albumin
PBS: Phosphate buffered saline
DTNB: 5,5′-dithiobis(2-nitrobenzoic acid)
TNB: 5-thio-2-nitrobenzoic acid
TCA: Trichloroacetic acid
EDTA: Ethylenediaminetetraacetic acid
EGTA: Ethylene glycol-bis(2-aminoethylether)-N,N,N′,N′-tetraacetic acid
Hsps: Heat shock proteins.
